# 

**Published:** 1867-04

**Authors:** 


### CONTENTS.

### ORIGINAL COMMUNICATIONS.

Enlightening and Directing public opinion in regard to the duties, responsibilities and requirements of Dental Practitioners........ 145
Animal Heat.—An Inaugural Thesis........ 148
A neglected communication........ 152
Presidents address to the Graduates of the Ohio College of Dental Surgery........ 156
Artificial Teeth........ 162
A large Alveolar Abscess........ 165
Dentinitis........ 166
How to Promote the Health of the Dentist........ 166

### PROCEEDINGS OF SOCIETIES.

Western Dental Society........ 168
Minutes of the twenty-third annual meeting of the Mississippi Valley Association of Dental Surgeons........ 170
The American Dental Association........ 178
The Association of the Colleges of Dentistry........ 179

### EDITORIAL.

Liberal prizes........ 185
In Memoriam —John S. Clark, D. D. S........ 186
Proceedings of Societies........ 187
The Association of the Colleges of Dentistry........ 188

